# Spontaneous intrapleural rupture of mediastinal teratoma in child

**DOI:** 10.4314/ahs.v22i3.33

**Published:** 2022-09

**Authors:** Ines Trabelsi, Marwa Zarrad, Manel Ben Romdhane, Sadok Boudaya, Fatma Khalsi, Khedija Boussetta

**Affiliations:** 1 Pediatric Department B, Children's Hospital of Tunis, Tunisia; 2 Surgery Department, Charles Nicolle Hospital, Tunisia

**Keywords:** Teratoma, tumor, mediastinum, child, rupture

## Abstract

**Introduction:**

Mediastinal teratomas are rare in children. Nevertheless, they represent the most frequent mediastinal germ cell tumor. Most often, they are discovered incidentally in older children or adolescents on chest X-ray. There are other signs of discovery but less frequent: chest pain, hemoptysis and signs of mediastinal compression. Rupture into pleural space, pericardium or tracheobronchial tree are exceptional.

**Case presentation:**

We report the case of 7-years old girl admitted for chest pain. The chest x-ray showed a mediastinal mass with calcifications and pleural effusion. Chest CT scan revealed a well limited heterogeneous anterior mediastinal mass with calcifications and a left pleural effusion. She underwent a median sternotomy and the tumor was completely excised. Histopathology confirmed the diagnosis of mature teratoma.

**Conclusion:**

Intrapleural rupture is a rare complication of mature teratoma. Calcifications on chest imaging in afebrile children with pleural effusion should be suspected of mediastinal teratoma.

## Introduction

Teratomas represent 10% of mediastinal tumors and 50 to 70% of mediastinal germ tumors^1^. They are mature in 80 to 88% and represent the most common benign type of tumors of embryonic origin^2^. In children, germ cell tumors (GCTs) account for approximately 25% of mediastinal tumors , including 60% of mature teratoma, 20% of mixed GCTs, and 20% of embryonal carcinoma^3^. Teratomas usually sit at the level of the antero-superior mediastinum and are often asymptomatic, which underestimates their real proportion^4^.

Teratomas rarely rupture in adjacent structures such as pleural space, pericardium or tracheobronchial tree and the skin with cystocutaneous fistula^5^,^6^.

We report an unusual case of intra-pleural rupture of mediastinal mature teratoma. This is a rare presentation of an uncommon pathology in children.

## Patient and observation

F.H, 7 years old girl, was admitted for a 2-month history of left-sided dragging chest pain. She had no pathological medical history and no significant past or family history of tuberculosis. She presented worsening pain during the last week, without fever, weight loss neither dyspnoea nor deterioration of the general state.

The physical examination of the patient showed a weight of 20 kg (-0.82SD), a height of 120 cm (-0.26SD), a normal body temperature and polypnea 35 cycles / min. There were decreased breath sounds on the left side with stony dull percussion note.

**Figure 1:**
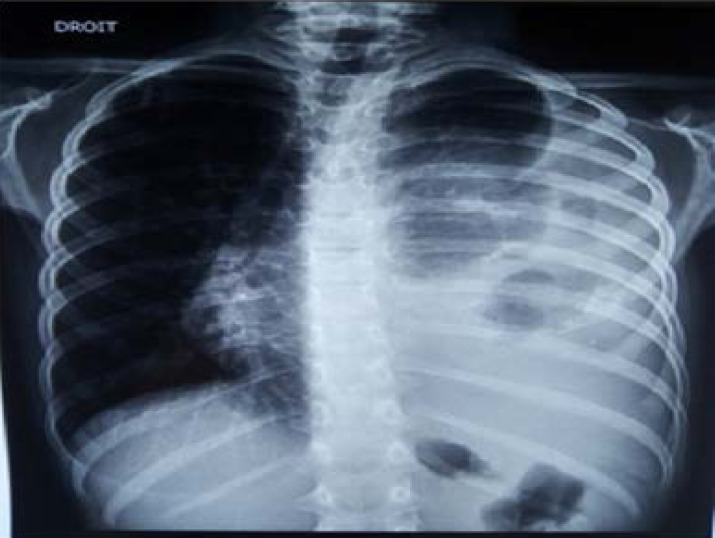
Chest X-Ray (P-A view) showing high density opacities occupying two-thirds of the left lung with cardiomediastinal shift to the right.

She was investigated thoroughly to eliminate possible causes that may give similar signs and symptoms. The chest X-ray showed high-density opacities occupying two-thirds of the chest, the site of irregular calcifications with deviation to the right of the trachea, heart and mediastinum (Figure 1).

**Figure 2:**
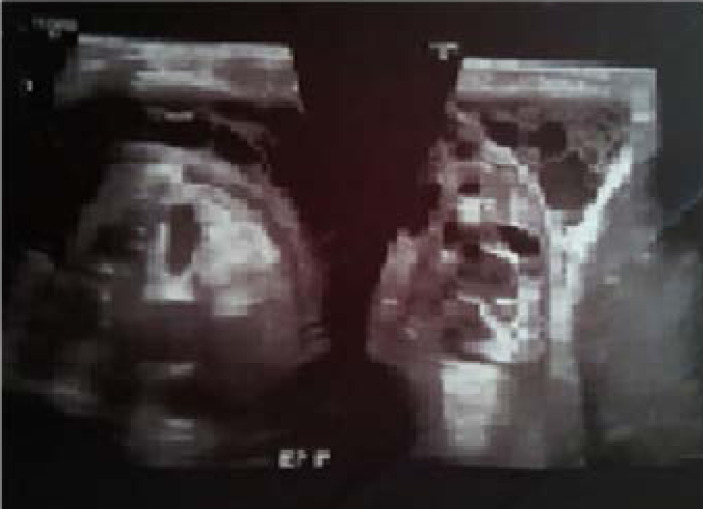
Chest ultrasonography showing multiple cysts occupying almost all of the left hemithorax associated with pleurisy

On chest ultrasonography, multiple cysts occupying almost all of the left hemithorax associated with pleural effusion were evident (Figure 2).

**Figure 3:**
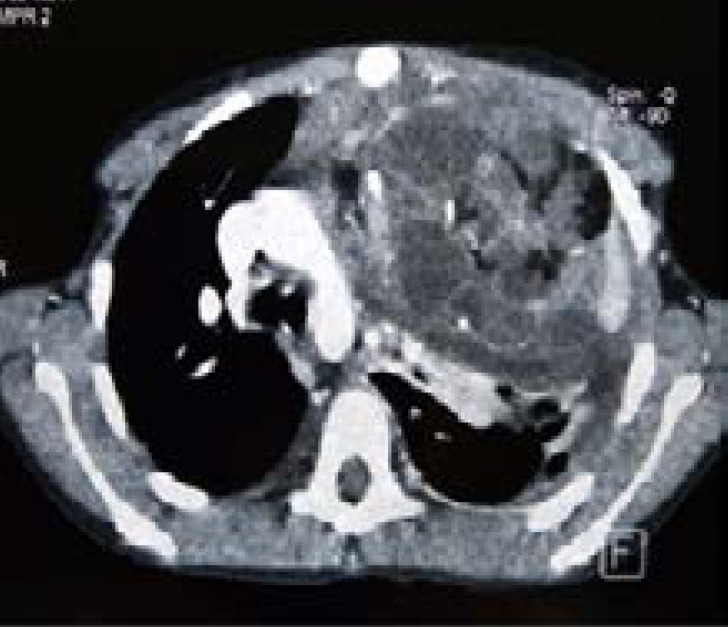
Chest CT showing heterogenous left-sided mass with many areas of calcifications

Laboratory explorations showed a WBC count of 12280/mm3 and a neutrophils count of 6990/mm3 and a serum C-reactive protein concentration of 32.72mg/l (normal range < 8 mg/l). Renal and liver functions tests were normal. Pyogenic abscess or empyema were unlikely because of absence of fever and laboratory test. The diagnosis of tuberculosis was suspected, but she had negative reaction to the Tuberculin skin test. The Sputum smear microscopy and culture were also negative for Zielh-Neelsen test. We considered an atypical intrapulmonary tumor or malformation. A contrast-enhanced computed tomography (CT) was performed, objectifying a heterogeneous mediastinal antero-left mass, well limited measuring more than 15 cm, many small areas of calcification and cavitation in the lower lobe of left lung associated with homolateral pleural effusion (Figure 3). Chest X-ray and CT scan findings were consistent with the diagnosis of calcified teratoma ruptured in the mediastinal pleura.

Biological assays: α fetoprotein (AFP) = 5.5 ng / ml and plasma beta-human chorionic gonadotropin (βHCG) <0.5 mUI / ml have rouled out malignancy.

The girl was operated on in the thoracic surgery department, by a total median sternotomy. During surgery, we saw a large tumor occupying the anterior mediastinum and almost the entire left thoracic cavity plating the lung in the costo-vertebral groove behind. The intervention consisted of a tumor resection with partial thymectomy. Biopsy and histology confirmed the diagnosis and identified a benign mature cystic teratoma consisting of different types of mature tissue. Our patient was followed up regularly at outpatients for 3 years. She was seen every 6 months to manage disease complications and screen for early recurrence. The follow-up visit included physical exam, laboratory testing and imaging. She was asymptomatic and the chest X-ray showed a well ventilated lung on the left, fetoprotein was normal one year after surgery. She remained well with no evidence of recurrence.

## Discussion

The mature teratoma is a germ cell tumor developed in the somatic stem cell and contain elements from all three embryological layers: ecto-, endo-, and mesoderm. It represents the most common benign type of germ cell tumors of the mediastinum. Teratomas are also found in sacrococcygeal, gonadal, retroperitoneal, cervicofacial and intracranial locations. Clinical behavior varies significantly by site and size^7^.

The discovery of the mediastinal teratoma is most often by coincidence. Patients with unruptured teratomas are often asymptomatic. The presence of symptoms depends on the size of the tumor or on the existence of a complication such as rupture towards the bronchus causing hemoptysis or trichoptysis wich is a pathognomonic symptom, towards the pleura causing pleural effusion with chest or back pain or towards the pericardium causing tamponade or pericadial effusion^8^. A literature review with the keywords: “ Teratoma, child, pleural effusion” on Pubmed revealed 5 cases of mediatinal teratoma with pleural effusion in children^9^–^13^. In our case, the tumor had ruptured into pleural space, and had caused sudden dyspnea in our patient while she has remained asymptomatic for a long time until hospitalisation.

The etiology of the perforation can be explained by local ischemia, related to the progressive growth of the tumor and is responsible for an adjascent necrosis, or an infection which can weaken the wall of the teratoma. On the other hand, cystic teratomas can produce proteolytic or digestive enzymes released by the pancreatic or salivary tissues. These cause a chronic inflammatory process in the wall of the tumor cyst, subsequently giving rise ta rupture or a fistula^14^.

Intrapleural rupture of mediastinal teratoma was commonly misdiagnosed as an encapsulated effusion or abscess. If in addition we have calcification and cavitation, the diagnosis of tuberculosis must be considered. In our context, in front of a chart of sudden dyspnoea with no fever, not too much elevated inflammatory markers in serum and calcifications in chest X-ray, pyogenic lung abscess or empyema seemed unlikely, but the diagnosis of tuberculosis was reasonable especially in high burden tuberculosis country such as Tunisia. However, tuberculosis workup in our patient was negative.

Moreover, mediastinal cystic or solid masses may arise in a variety of benign and malignant disorders. Lymphoma is a common etiology of mediastinal masses in children. It arise from the anterior or middle mediastinum and associate cardiopulmonary disorders. Neuroblastoma originate from the posterior mediastinum, rarely producing airways obstruction. Other possible differential diagnoses of thoracic opacities in children must be considered including hydatidosis lung, thymoma, germ cell tumor, pulmonary hamartoma, bronchogenic cyst, adenomatoid cystic malformation and intrapulmonary cystic lymphangioma. Imaging is the investigation of choice to guide the diagnosis^15^.

The chest x-ray is an important exam that leads to the discovery of most mediastinal abnormalities^16^. With the presence of both fat and calcification, diagnosis of an unruptured mature teratoma is not difficult. However, when this type of tumor ruptures, radiologic findings may be similar to those for a malignant infiltrative tumor.

Computed tomography CT is the investigation of choice where teratomas appear as anterior mediastinal masses with fluid component, calcification, soft tissue and fat attenuation. It's good exam for studying the invasion of the surrounding tissues but it isn't the best to show the adhesions^15^. It shows a circumscribed tumor with an often thick capsule, sometimes calcified, which increases with the injection of contrast product.

However, the final diagnosis can only be made after surgical intervention.

The treatment remains surgical. The median sternotomy is the most used for tumors with bilateral overflow^17^, but some authors prefer posterolateral thoracotomy or anterior thoracotomy on the lower fold of the breast, for tumors with unilateral overflow. Median sternotomy associated with thoracotomy has been described for large tumors^11^.

Biopsy is important in order to study the tissue component. Specific treatment depends on the type of teratoma. It generally includes surgical removal of tumors for matutre teratoma.

A combined approach of surgery and chemotherapy has been recommended for immature mediastinal teratomas^18^. After a complete surgical intervention, the prognosis for mature mediastinal teratomas is excellent.

## Conclusion

Mature teratomas of the mediastinum are rare. The clinical symptomatology is not specific and is most often manifested by signs of complications. Therefore, it can be difficult to diagnose especially in early stage. They should be considered in the differential diagnosis of thoracic opacity. They are often recognized by CT.

The concern for the surgeon is to carry out the complete excision of the tumor, and for the pathologist to seek an immature squad within this tumor, the presence of which determines the prognosis and the complementary treatments.
